# Food Purchasing Behaviors of a Remote and Rural Adult Solomon Islander Population

**DOI:** 10.3390/foods8100464

**Published:** 2019-10-10

**Authors:** Charis Bottcher, Steven J. R. Underhill, Judith Aliakbari, Sarah J. Burkhart

**Affiliations:** 1 School of Health and Sport Sciences ML41, University of the Sunshine Coast, Locked bag 4, Maroochydore DC, Queensland 4558, Australia; 2 Australian Centre for Pacific Islands Research, University of the Sunshine Coast, Locked bag 4, Maroochydore DC, Queensland 4558, Australia; Sunderhi@usc.edu.au; 3 School of Natural Resources and Applied Sciences, Solomon Islands National University, Honiara, Solomon Islands; 4 The Hilltop Training Institute, Auki, Malaita, Solomon Islands; jaliakbari9@gmail.com

**Keywords:** nutrition, food choice, Pacific Islands, Auki, Malaita

## Abstract

The aim of this study was to investigate the food purchasing behaviors of an adult Solomon Islander population within a transitioning food system in Auki, Malaita. Food purchasing behavior measures included; venue type and transportation for purchasing food, previous day expenditure on food purchases, number of weekly shopping experiences for store foods (generally long-life shelf and frozen items) and fresh foods (such as fruits and vegetables and fresh fish) and the importance of factors (i.e., price) on purchasing decisions. One hundred and thirty-three adults (aged 18 to 74 years; female: 63%, males: 37%) completed an interviewer administered questionnaire during December 2018. Food items were primarily sourced from Auki markets (*n* = 70) and stores (*n* = 40). Food purchasing differed between fresh and semi-perishable foods (store food). Participants reported similar shopping experiences for store food and fresh food (M = 3.87 and M = 3.25 times a week, respectively) and spending between $1 and $200 (M = $56.12) Solomon Island dollars on food in the previous day. The most reported purchased item was white rice (*n* = 117, 88%), with taste, freshness and family preference the most important factors reported as influencing food purchasing choices. While our findings are from a small sample in Auki, further research could build upon this work by investigating food purchasing behaviors at other times of the year, and more widely in the Solomon Islands and greater Pacific region.

## 1. Introduction

There is increased interest in the ways that individuals and communities in remote and transitioning food systems source food. Many traditional food systems around the world, including in the Pacific Islands, are in the process of nutrition transitions. These transitions are seen as changes within the populations energy expenditure, dietary intake and patterns as food systems transition from traditional local food supply to modern food systems based largely on imported processed foods generally higher in fat, salt and sugar, potentially impacting food security [1,2,3].

The rates of diet-related noncommunicable diseases (NCDs), including obesity and Type 2 diabetes in the Pacific Islands countries and territories (PICTs) are some of the highest worldwide, yet the trajectory of this epidemic is predicted to continue increasing [4,5,6]. Changes in diet and the food environment are considered the main factors for the regions rise in NCDs [2,5,7,8]. Given dietary intake directly contributes to health status, in this region where increasing quantities of imported foods are becoming readily available, it is increasingly important to understand the impact these changes are having on food purchasing behaviors and drivers of food purchasing decisions, however there is currently limited literature in this area.

Solomon Islands, a group of six main islands and over 900 small islands lie to the north east of Australia. Population growth, urbanization and climate change in the Solomon Islands, as in other PICTs, has resulted in an accelerated nutrition transition from a traditional food system based on locally sourced fresh fruits, vegetables, tubers and fish to a western-based food system based largely on highly processed foods that are generally high in fat, salt and/or sugar [9,10,11,12]. This has led to an increased demand for imported foods such as canned meats, instant noodles, cereals, rice and sugar-sweetened beverages threatening local food security [2,7,12].

Previous studies on food environments and health status have highlighted the importance of identifying factors that influence dietary behaviors and food security, including food purchasing behaviors in disadvantaged locations as an important step to ensure interventions and policy changes are informed by local evidence [13,14,15]. Food purchasing behaviors are influenced by food accessibility and food availability in the local community and neighborhood [15,16]. Several factors have been identified previously to influence household purchases in low-income settings [14,16,17,18], including affordability/cost [2,14,16,17,18], ability to transport food from the place of purchase to home [12,19], and seasonal changes [19]. Although Solomon Islanders have been reported to understand the health importance of local foods (traditional diet) and that processed foods are likely unhealthy, there is currently limited research related to how food is sourced in remote coastal communities [4,12]. The aim of this study was to investigate the food purchasing behaviors of a general adult Solomon Island population within a transitional food system.

## 2. Materials and Methods

### 2.1. Study Design and Setting

**Figure 1:**
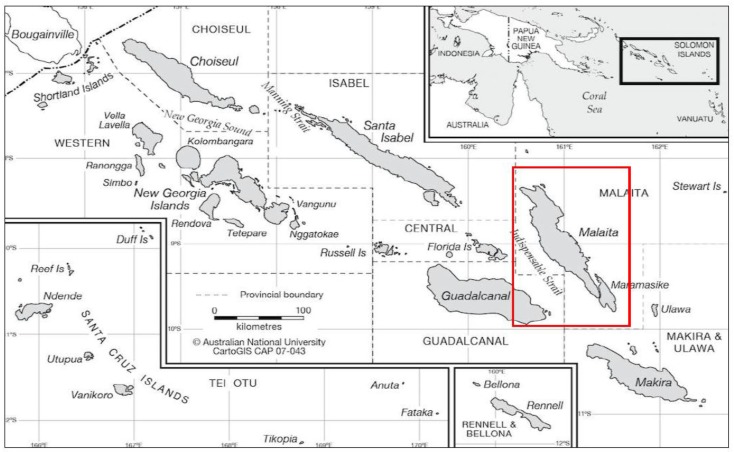
Map of the Solomon Islands. Location of Malaita Island (indicated in the red box) within the Solomon Islands archipelago (Map source: CartoGIS Services, College of Asia and the Pacific, The Australian National University, Australia, 2018) [21].

An observational descriptive cross-sectional study design was used to investigate the food purchasing behaviors of adults living in Auki, Malaita, Solomon Islands. Auki, the capital, main port and business area of the island of Malaita has a population of approximately 5105, compared to Solomon Islander population of over half million people [12,20] (Figure 1). Being a remote, coastal and rural location, Auki Malaita provides an interesting environment to investigate food related behaviors in a changing food system and current nutrition transition. Ethical approval for this study was provided by University of the Sunshine Coast (Ethics Application code S181248) and the study was conducted in compliance with the guidelines presented in the Declaration of Helsinki.

### 2.2. Participants and Recruitment

During a thirteen-day period in December 2018, four Australian researchers working with four local Solomon Islands translators (the research team) randomly approached adult residents of Auki at locations in and around Auki town including markets, stores and in open public spaces informing them of interview purpose and asking if they would like to participate. Inclusion criteria was participants aged 18 years or older and a current permanent resident of Auki (Auki town center and surrounding villages of the Auki district). Exclusion criteria included being unable to or refusing to provide verbal consent and those who do not purchase food. Verbal consent was received and a research project information sheet with information on data collection and use was available for participants.

### 2.3. Tools and Measures

The interviewer administered questionnaire included questions on demographic characteristics and food purchasing behaviors, and was based on tools previously used in similar contexts of low-income or low socio-economic status (SES) areas, although predominantly in urban locations [17,18,22,23]. Demographic measures included; sex, age, location, accessibility to own food garden, education level, household number, job/occupation, and sources used to access food. Total net income was not measured as participants were uncomfortable answering this question during the pilot of the tool undertaken in Auki. Food purchasing behavior measures included; the venue type used for purchasing food (store, market, roadside vendor, other) transportation used to travel to purchase foods, previous day expenditure on food purchases (in Solomon Islands dollars (SBD)), and the number of times food shopping was completed each week for store foods (generally packaged long-life shelf and frozen items) and fresh foods (such as fruits and vegetables and fresh fish).

Participants were also asked to report how influential the factors; taste, price, freshness, easy preparation and family preference, were on food purchasing behaviors on a scale from one (not important) to three (very important), with an option to select “don’t know” as a response. Foods purchased were based on participant responses of foods and beverages eaten in the last 24 h time period at home or outside the home for breakfast, lunch, dinner and snacks which were then categorized into specific food groups (cereals, white tubers and roots, vegetables, fruits, meat, eggs, fish and other seafood, legumes/nuts/seeds, milk/milk products, oils/fats, sweets and spices/condiments/beverages).

Throughout the interview participants were prompted to complete the questionnaire using a prepared script, with responses recorded on a paper-based questionnaire. Each interview took approximately 25 minutes to complete. Translators interviewed participants in English, Pidgin or the local dialect based on the participants preference and recorded all responses in English. Any responses that were unclear were discussed with the research team to clarify correct translation. Both researchers and translators were provided with training including an explanation of the combined questionnaires, role play for approaching and phrasing questions to participants, and how to complete the questionnaire form. A research team meeting was held before each data collection session to clarify the procedure and cultural considerations for data collection.

### 2.4. Analysis

Analysis was completed in IBM Statistical Package for Social Sciences (SPSS version 24, SPSS Inc., Chicago, IL, USA, 2016) using descriptive analysis for observational data. Associations between variables were calculated with Mann-Whitney U, Kruskall-Wallis analysis of variance tests, and the Chi Square test. Where size assumptions were not met for Chi Square, the Fischer’s Exact test was used. Data is presented as the sum, proportion and/or mean, median and SD for demographic characteristics and food purchasing behaviors. The grouping of the foods purchased is based on the food groups used in the Food and Agriculture Organization of the United Nations (FAO) dietary diversity assessment tool [24].

## 3. Results

### 3.1. Demographic Characteristics

A total of 133 participants were interviewed, with participants aged between 18 and 74 years, and a higher proportion of females (63%) to males (37%). Approximately half of those interviewed resided in Auki town, with out of town participants representing numerous rural communities and villages. Most participants (66%) had access to a home garden, with relatively equal representation between those in paid employment to those self-classifying themselves as unemployed. Those in paid employment represented more than 25 job types. A total of 13.6% of those surveyed were associated with the food system (i.e., 6.8% market vendors, 3.8% retail, and 3.0% farming (home and subsidiary)) (Table 1).

### 3.2. Food Purchasing Behaviors

#### 3.2.1. Sources of Food

Participants reported accessing fresh food from a range of sources. Over half reported mainly using the Auki markets (57%) to source fresh food, however, this was followed by sourcing fresh food from their own food gardens (30%) and a combination of sourcing fresh food from the Auki markets and their own food gardens (13%) (Table 2).

All reported food items were primarily purchased from the Auki markets (*n* = 70), stores (*n* = 40), and a combination of store and other food venue (*n* = 10). Roadside vendor and a combination of Auki markets and another food venue was reported by only two participants respectively. Nine participants did not indicate the venue/s they purchase foods from.

#### 3.2.2. Reported Food Expenditure

Participants reported spending between $1 and $200 SBD (Mean = $56.12 SBD, SD = 47.63) on food purchases in the previous day (at the time of this study 1 SBD was equivalent to 0.12 USD/0.11 EURO) [24]. Employed participants were more likely to report spending a significantly greater amount of money on food in the previous day (Mean = $63.85 SBD, median = $50.00 SBD) compared to unemployed participants (Mean = $47.81 SBD, median = $30.00 SBD) (*p* = 0.004). Fifteen participants did not provide a price range for food expenditure.

#### 3.2.3. Number of Reported Food Shopping Experiences

Participants reported a similar number of shopping experiences a week for both fresh food (Mean = 3.25) and store food (Mean = 3.87) (Table 3).

The most common forms of transportation reported by participants when travelling to purchase food was walking (55.3%, *n* = 68), public bus (16.3%, *n* = 20), car (11.4% *n* = 14), boat/canoe (8.9%, *n* = 11), public truck (3.3%, *n* = 4), taxi (2.4%, *n* = 3). Some participants reported a combination of transport modes, including walking and bus (1.6%, *n* = 2) and walking and boat/canoe (0.8%, *n* = 1). Ten participants declined to report transportation type used when travelling to purchase food.

#### 3.2.4. Types of Foods Purchased in the Previous 24 Hours

Food groups and associated items purchased by greater than 10 participants are shown in Table 4. Products purchased by less than 10 participants in total included: Cereal foods (navy biscuits, white flour, coco biscuits, savory crackers, Weetabix^TM^ and popcorn), Tubers and roots (hot chips, breadfruit and yam), Vegetables (onion, taro leaf, mangrove root, lettuce, pumpkin tips, watercress and cassava leaf), Fruit (lemon, lime, starfruit, guava, mandarin, local apple, potera, orange juice, pomelo, soursop, avocado and local cherry), Meat and eggs (pork, deep fried fish, sausage, chicken and beef steak), Nuts (kat nus and peanut butter), Dairy (ice cream), Fats (palm oil, other cooking oil or vegetable oil, and butter), Discretionary (sweet drinks, donuts, chips, candy, milo), Condiments, Spices and Beverages (various types of sauces, ginger, garlic, flavoring and alcohol). Females were more likely to report having purchased items from the nuts group (*p* = 0.046) and fats group (*p* = 0.006), while those who lived outside of Auki town were more likely to report purchasing items from the condiments group (*p* = 0.019). No other significant relationships between purchased items and location, sex or age were identified.

#### 3.2.5. Influences on Food Purchasing Behaviors

**Figure 2:**
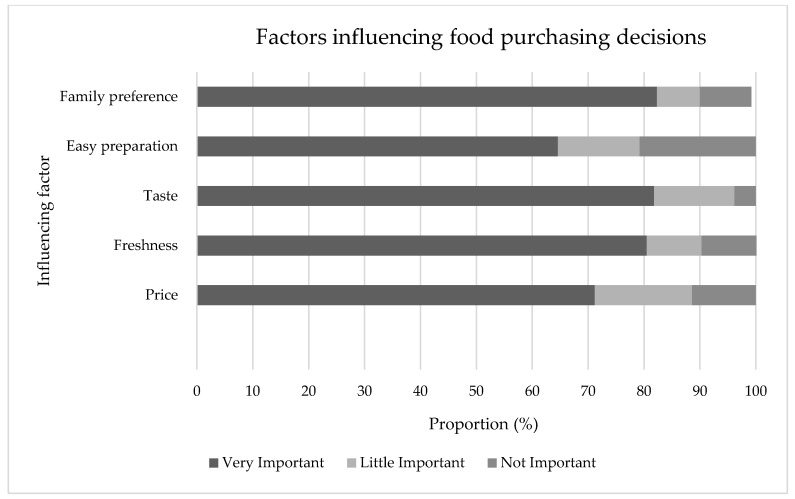
The level influence of price, freshness, taste, easy preparation and family preference on food purchasing behavior of participants.

Similar numbers of participants reported taste (*n* = 108, 81.8%), family preference (*n* = 107, 82.3%) and freshness (*n* = 107, 80.5%) as a very important influence on food choice (Figure 2). There were no associations between those participants who lived in town versus out of town or sex and any of the factors influencing food purchasing decisions.

## 4. Discussion

This study investigated the food purchasing experiences of an adult Solomon Island population. Given the transition from subsistence food production and the increased rates of nutrition related health consequences seen in this population [11,12,20,25,26], understanding food purchasing behaviors is of importance. Our study population, noting the exclusion of youth less than 18 years old in the survey sampling, was partially consistent with population demographics reported in the last census for Malaita [20]. The overall mean age (years) of adult survey participants was 37 years, with the mean age of males 39.63 and females 35.67 years, compared to 58% of adult population in Malaita being aged 25–59 years [20]. Mean household size of was 6, consistent with the Auki census household size of 5.8 [20]. Education levels in the sample cohort differed from the Malaita census data. This study found that 9.8% of the adult population had no schooling and 33.1% primary school-level education, whereas the census data reported 27% with no schooling and 55% having received up to primary school education [20]. There was also a notable difference in the gender ratio, with 63.2% of survey participants being females, compared to a 49.7% female populational representation. Much of this disparity can be accounted for. Auki has a higher level of education and greater employment participation than in the rest of Malaita [20]. The high portion of females in our sample population reflects the fact that in Solomon Island culture women are primarily responsible for food shopping, with males more likely to be involved in paid employment.

Food purchasing behavior in Auki differed between fresh and semi-perishable (store food). This frequency of fresh food purchasing reflected the observed trading activity at the Auki market, with most of the vendor participation concentrated in the latter part of the week (Thursday to Saturday). In the case of semi-perishable store food, which are generally tinned or packaged with a long shelf life, there was an interesting trend toward more frequent purchasing. It may be that these items are considered more convenient, or portable and are therefore purchased more frequently. Additionally, these items tend to be heavier, and therefore it may be more difficult to buy quantities of these foods, particularly if an individual’s main form of transportation is walking. A study investigating definitions of food access and availability identified that the safety of walking routes was also an important factor [27]. We found that 55% of our population reported walking as their main form of transport to purchase foods, with this form of transport having previously been identified as a factor limiting large purchase amounts or with reduced accessibility in seasonal changes [15,19].

We found that the most common source of food reported by participants was from the Auki market, followed by home gardens. Previous studies have noted that subsistence-based gardening has been an important contributor to fresh produce intake amongst the community in Solomon Islands and Malaita [4,11,12], so there is also likely to be a strong connectivity between these two food sources. The most frequently purchased perishable crops from the Auki market were sweet potato, cabbage, tomato and cucumber. The purchasing of higher-value vegetables (i.e., tomato, cucumber, and to a lesser extent capsicum, eggplant, snake bean and spring onion) is likely to reflect the fact that these crops can be more difficult to produce within a subsistence-based home-garden due to the accessible of planting material, and the need for more intensive production practices. The purchase of traditional crops from the market, particularly sweet potato, cassava and plantain, all of which are commonly grown within subsistence-based home gardens, is more difficult to explain. It is possible that participants visit the markets to purchase items that they do not have ready access to in their own garden, or in the case of traditional crops insufficient quantity to support a specific need (i.e., customary or family event). More speculative, is the possibility that the occasional purchase of traditional crops from the market, ensures home gardens are not fully depleted maintaining a reserve source of household food. With most participants having access to a home garden, this may explain why fresh foods, particularly fruits and vegetables, were purchased less often. We did not ask participants to report what foods they access from a garden, or if any sharing/trading occurred in their community. Ready-made items (i.e., fried fish and sweet potato chips, cassava pudding) were also available in the market at the time of this study and it is possible that these were purchased while participants were working or socializing in the marketplace. Future work is required to better understand what foods are being sourced, and where from as this may assist with the planning subsequent research projects and understanding influences on food consumption behaviors.

The most frequently purchased food items in this study were white rice, tea, cabbage, sugar, tinned fish, and salt. Of these items, only cabbage and most likely tinned fish were locally sourced items from Malaita or Solomon Islands and could be categorized into food recommended for consumption in one of the three main food groups according to the Pacific guidelines for a healthy diet and lifestyle [28,29]. While our purchasing data does not necessarily equate to consumption, it is interesting to note that a number of these items were not traditional, local foods (white rice, tea, sugar, salt), and high consumption of these foods may be linked to adverse health outcomes [2,6]. In the Solomon Islands traditional dietary carbohydrates include sweet potato, taro and cassava (tubers and roots) [2,11,12]. We found that in comparison to reported numbers of participants purchasing white rice, the traditional carbohydrate sources of sweet potato and taro and cassava had a much lower reported purchase rate. This may reflect access to local tubers and root crops in home or community gardens, and/or a preference for rice based on price per serve, shelf life and ease of preparation. A price comparison collected at the same time as this study was undertaken found that 1kg bags of rice compared in price to bunches of the traditional carbohydrate energy foods [29]. Another study in Malaita noted participants choice for imported foods, such as rice and noodles factored into three main reasons: climate change (associated to increased disruption to local production), traditional family roles changing and urban migration [12]. Given potential health implications of overconsumption, it would be of interest to understand how items such as sugar and salt are being used. For example, a study in Kiribati (in the Micronesia region of the Pacific Islands) found participants had a high consumption of sugar, and that it was common practice to add sugar to most meals [6]. In a recent review of fat, sugar and salt in Pacific Islander diets [30], the paucity of information on associated consumption levels and dietary behavior was noted.

Tinned fish was one of the most frequently purchased items, and the most purchased item from the meat and eggs food group. There may be several reasons for this finding, including a preference for tinned fish, its comparatively low perishability, cost and the possible lack of alternative protein sources [7,11,23]. Fishing is an important occupation and contributor to the local economy and more income can be derived from exporting locally caught fish than selling this locally [23]. Locals may therefore prefer to sell caught fish, and instead rely on tinned fish for personal consumption. Tinned fish is convenient, has a long shelf life, and does not require refrigeration prior to opening. According to the 2009 household census only 1% of households in Malaita had a refrigerator and only 3% had access to main-grid electricity [20]. Our parallel study [29], found that tinned fish was less expensive than imported frozen meats such as chicken, beef and pork or canned meat products in Auki, and that varying flavors of tinned fish are available, potentially increasing flavor diversity. Additionally, previous studies have reported that non-fish animal source-foods are rare in the diets of Solomon Islanders [4,11]. A recent FAO report on the Asia-Pacific region [2] notes there has been a rise in consumption of animal source foods, however much smaller increases in Pacific region. Small numbers of participants reported purchasing dairy products which is not surprising given its limited availability in Auki [29], with low reported intake in Solomon Islands and more broadly across the Pacific Island region [4,6,11].

The influences of taste, freshness and family preference appeared to be more important to our sample. This is not surprising given that taste has previously found to be of importance in general [31,32], and to the people of Malaita when purchasing foods from stores, unlike fresh produce and meats where taste was not cited as an influencing factor [12]. Many of the foods found in store are high in salt and/or fat, and/or sugar, making them more palatable in comparison to traditional foods which may be perceived as blander.

Our findings suggest that price, while important, was not the primary factor influencing food purchasing behavior in Auki. This finding is of interest given high rates of unemployment. Participants spent an average of $56.12 SBD (equivalent to $6.82 USD/$6.16 Euro) on food during the previous day [33]. With most participants undertaking one to three shopping events per week, this equates to around $673 SBD (equivalent to $81.77 USD/$73.82 Euro) of expenditure on food per month [33]. This study did not collect household income levels, however to put this into perspective, it has been estimated that monthly household income in Solomon Islands ranges from $55 to $46,100 SBD (equivalent to $6.68 to $5601.15 USD/$6.03 to $5057.33 Euro), with a median of $1910 SBD per month (equivalent to $232.06 USD/$209.53 Euro), varying between village [23] (1 SBD equals 0.12 USD/0.11 Euro) [33]. We did not collect household income but recognize that this would be of interest, particularly if there is a relationship to the types of foods that are purchased. While no studies have directly linked household income to food purchasing behaviors in this setting, another study undertaken in Malaita reported that consumption of some foods (i.e., “special foods” like tinned meat and tinned fish) are linked to cost [18]. Given that participants in this study reported accessing food from various sources, it is possible that the money used to purchase food can be utilized for preferred food items, complemented by foods that may be accessed for no, or little cost (for example, from home gardens and the ocean). Further research investigating the role of household income, food cost and purchasing behaviors, as well as mapping food sources, would be of value in this setting and could inform potential public health interventions.

Freshness may be considered important to our sample as a parallel study in Auki participants reported a preference for local food [34]. Another study undertaken in Malaita reported that vegetables, fruits and fresh meats were perceived as foods to provide energy and protect against disease [12]. Freshness may also be used as a qualitative measure of food safety risk and nutritional benefit. Family preference may be an important influence given household number size and observation from previous study [12]. The concept of Wantok (meaning “one talk”), defined as an extended family of people speaking the same language/dialect who take care of each other, is used in Solomon Islands [11]. This includes providing one meal, usually comprised of less expensive, but filling foods (i.e., rice, root crops, noodles) to feed a large group of people [11,12]. Participants in our study reported similar household numbers compared to that published previously [23], however, we did observe a higher maximum household size. While we did not find any associations between education level and influences on shopping behaviors, other studies have found that higher education qualifications were the main factor associated with having healthier dietary behaviors and health status [4,13,18]. Our sample size may have been too small and homogenous to identify any differences. Further research to understand influences on food purchasing decisions, particularly regarding preference for local fresh food, but purchasing of imported packaged products would contribute to the very limited literature in this area. Additionally, school food and nutrition education has been reported as being limited in some areas of the Pacific region including Solomon Islands [35], and further research into education level, particularly food and nutrition education, and the influence this has on food purchasing behaviors is warranted.

### Limitations

Our project has several limitations. We needed to adapt the tool used to be suitable for our setting, resulting in several changes during the pilot stage. The tool was effectively validated, predicated on appropriate sampling intensity and frequency. The development of a tool suitable for the Pacific region may assist similar research in the future. As our sample size is small and we only collected data from Auki, our findings may not be representative of the greater Malaita or Solomon Islands region. Our data was collected at one time point which limits the transferability of our findings over different seasons and periods throughout the year. We had a largely female based population, however the traditional gender roles in Solomon Islands dictate that males are generally responsible for earning the money, while women are responsible for food shopping, cooking, cleaning. In addition, not asking participants who in the household is responsible for purchasing decisions could be a limitation in understanding of overall household purchasing decisions. In employment findings, of the participants that listed “other” as a job choice the most common description of these was housewife which could also be described as looks after/takes care of family. One could argue that this is in fact also being unemployed, as in not bringing in an income to the family, which could also increase the actual numbers of unemployment. However, during data collection participants were asked for “occupation”, potentially cultural context of family role of taking care of the home could be accepted as occupation. Further specifications on what occupation refers to should be offered in future studies.

## 5. Conclusions

Our study provides preliminary insight into the food purchasing behaviors of members of a rural, coastal and remote community in the Solomon Islands. While food purchasing behaviors do not necessarily equate to consumption, given current nutrition transitions and the high incidence of diet-related NCD’s observed throughout the Pacific region, further research investigating influences on these behaviors, particularly sociodemographic characteristics, and mapping sources of food are important steps towards understanding drivers of food security, nutritional status and health.

## Figures and Tables

**Table 1:** Participant demographic characteristics.

Demographic Characteristic	
Sex	*n (%)*
Male	49 (36.8)
Female	84 (63.2)
Age (years)	mean, (SD)
Overall mean	37.1 (12.19)
Male	39.6 (13.02)
Female	35.7 (12.19)
Participant home location	n (%)
In Auki Town	52 (39.1)
Out of Auki Town (villages: Ambu, Fasitoro, Finkawi, Kawi, Kilfui, Kilusakwalo, Kunu, Lilisiana, New Kaloka, Numerula, Sikitae, Talakali, Other)	81 (60.9)
Reported no. people in household	mean (range)
Current household size	6 (1–20)
Maximum household size	7 (1–30)
Access to own garden	*n %*
Yes	86 (65.6)
No	45 (34.4)
Education level	*n* (%)
Primary years 1–6	44 (33.1)
Secondary Form 1–3	25 (18.8)
Secondary Form 4–6	31 (23.3)
Secondary Form 7	3 (2.3)
University	13 (9.8)
Technical	4 (3.0)
Unable to attend	13 (9.8)
Job Type ^^^	*n (%)*
Total Employed	65 (49.2)
Teacher	9 (6.8)
Market Vendor	9 (6.8)
Retail	5 (3.8)
Police	3 (2.3)
Farm—Home	2 (1.5)
Farm—Subsidiary	2 (1.5)
Other *	35 (26.5)
Total Unemployed	67 (50.8)
Housewife/Looks after family/Takes care of family	8 (6.1)
Retired	4 (3.05)
Student	4 (3.05)
Activity not defined	51 (38.6)

Total sample population = 133. * Other includes; baker, builds boats, canteen shop owner, carpenter, civil engineer, correction service, council of Malaita, electrician, government job, housekeeper, library, market manager, ministry of commerce, nurse, pastor, radio frequency engineer, sales & marketing officer, security officer, self-employed, shop manager, taxi driver, youth organization. ^ 1 missing response, *n* = 132.

**Table 2:** Reported sources where participants access fresh food.

Fresh Food Sources	*n (%)*
Market	76 (57)
Own garden	30 (22.5)
Garden & market	13 (9.8)
Store	5 (3.8)
Market & store	3 (2.3)
Ocean	2 (1.5)
Market & ocean	2 (1.5)
Market, ocean & store	1 (0.8)
Own garden & store	1 (0.8)

**Table 3:** Number of shopping experiences per week for fresh and store food.

Number of Times a Week	Fresh Food (Total *n* = 130) *n (%)*	Store Food (Total *n* = 127) *n (%)*
1 a week	31 (23.8)	29 (22.8)
2 a week	29 (22.3)	21 (16.5)
3 a week	34 (26.2)	25 (19.7)
4 a week	5 (3.8)	3 (2.4)
5 a week	1 (0.8)	4 (3.1)
6 a week	3 (2.3)	1 (0.8)
Daily	27 (20.8)	44 (34.6)
Mean (SD) number of shops a week	3.25 (2.18)	3.87 (2.47)

**Table 4:** Type of food items purchased based on sex and location (highest reported items from all food groups).

Food Group and Item	Number and Proportion of All Participants Who Reported Purchased Item				
Cereal		Gender *n* (%) ^#^		Location n (%) ^^^	
	TOTAL *n* (%) *	Female	Male	Auki town	Out of Auki town
White rice	117 (88)	74 (63.2)	43 (36.8)	43 (36.8)	74 (63.2)
Bread	39 (29.3)	28 (71.8)	11 (28.2)	13 (33.3)	26 (66.7)
Noodles	33 (24.8)	23 (69.7)	10 (30.3)	10 (30.3)	23 (69.7)
Breakfast cracker	31 (23.3)	22 (71)	9 (29)	12 (38.7)	19 (61.3)
Tubers and roots					
Sweet potato (white)	55 (41.4)	36 (65.4)	19 (34.5)	19 (34.5)	36 (65.4)
Sweet potato (other)	42 (31.6)	28 (66.6)	14 (33.3)	12 (28.6)	30 (71.4)
Taro	20 (15)	12 (60)	8 (40)	12 (60)	8 (40)
Cassava	20 (15)	14 (70)	6 (30)	8 (40)	12 (60)
Vegetables					
Cabbage	90 (67.7)	59 (65.5)	31(34.4)	35 (38.9)	55 (61)
Tomato	41 (30.8)	28 (68.3)	13 (31.7)	18 (43.9)	23 (56.1)
Cucumber	36 (27)	28 (77.8)	8 (22.2)	14 (38.9)	22 (61.1)
Capsicum	25 (19)	21 (84)	4 (16)	12 (48)	13 (52)
Eggplant	22 (16.5)	19 (86.3)	3 (13.6)	7 (31.8)	15 (68.1)
Snake bean	21 (16)	14 (66.6)	7 (33.3)	5 (23.8)	16 (76.2)
Pumpkin	21 (16)	16 (76.2)	5 (23.8)	5 (23.8)	16 (76.2)
Spring onion	20 (15)	13 (65)	7 (35)	6 (30)	14 (70)
Fruit					
Banana	33 (24.8)	22 (66.6)	11 (33.3)	7 (21.2)	26 (79)
Papaya	28 (21)	20 (71.4)	8 (32)	10 (35.7)	18 (72)
Watermelon	27 (20.3)	23 (85.2)	4 (14.8)	12 (44.4)	15 (55.6)
Pineapple	26 (19.5)	20 (76.9)	6 (23)	8 (30.8)	18 (69.2)
Coconut	23 (17.2)	13 (56.6)	10 (43.5)	7 (30.4)	16 (70)
Mango	20 (15)	14 (70)	6 (30)	4 (20)	16 (80)
Plantain	18 (13.5)	12(66.7)	6 (44.4)	5 (27.8)	13 (72.2)
Meat and eggs					
Tinned fish	76 (57.1)	50 (66)	26 (34.2)	28 (36.8)	48 (63.2)
Fresh fish	39 (29.3)	27 (69.2)	12 (31)	16 (41)	23 (59)
Eggs	18 (13.5)	13 (72.2)	5 (27.8)	6 (33.3)	12 (66.7)
Seafood crab	11 (12)	7 (58.3)	4 (36.3)	5 (45.4)	6 (54.4)
Nuts **					
Ngali nut	37 (27.8)	25 (67.63)	12 (32.4)	11 (29.7)	26 (70.2)
Peanuts	15 (11.3)	10 (66.7)	5 (33.3)	6 (40)	9 (60)
Dairy					
Milk powder	18 (13.5)	12 (66.47)	6 (33.3)	3 (16.7)	15 (83.3)
Fats ***					
Coconut cream	38 (28.6)	30 (79)	8 (21)	16 (42)	22 (58)
Coconut milk	37 (28)	24 (65)	13 (35.1)	12 (32.4)	25 (68)
Coconut oil	18 (13.5)	13 (72.2)	5 (27.8)	4 (22.2)	14 (77.8)
Peanut oil	13 (9.8)	10 (77)	3 (23)	4 (31)	9 (69.2)
Discretionary					
Sugar	80 (60.1)	49 (61.2)	31(39)	29 (36.2)	51 (64)
Cake	22 (16.5)	16 (72.7)	6 (27.2)	5 (22.7)	17 (77.2)
Soft drink	22 (16.5)	15 (68.1)	7 (31.8)	10 (45.4)	12 (54.5)
Flavored ice block	20 (15)	16 (80)	4 (20)	7 (35)	13 (65)
Sweet bun	15 (11.3)	9 (60)	6 (40)	3 (20)	12 (80)
Sweet biscuit	12(9)	9 (75)	3 (25)	6 (50)	6 (50)
Condiments ^^^^					
Tea	95 (71.4)	60 (63.1)	35 (36.8)	35 (36.8)	60 (63.1)
Salt	69 (52)	51 (73.9)	18 (26)	20 (29)	49 (71)
Soy sauce	26 (19.5)	20 (76.9)	6 (23)	7 (27)	19 (73)
Coffee	18 (13.5)	15 (83.3)	3 (16.7)	1 (5.6)	17 (94.4)
Curry	11 (82.7)	7 (63.6)	4 (36.3)	6 (54.5)	5 (45.4)

* Proportion calculated from total n of 133, ^#^ proportion calculated from total n of the participants who reported purchasing this item, ^^^ proportion calculated from total n of the participants who reported purchasing this item. ** Females were more likely to report purchasing items from the nuts groups (*p* = 0.046), and items from the fats group *** (*p* = 0.006). ^^^^ Those who lived outside of Auki town were more likely to report purchasing items from the condiments group (*p* = 0.019).
